# The spinal stenosis pedometer and nutrition lifestyle intervention (SSPANLI) randomized controlled trial protocol

**DOI:** 10.1186/1471-2474-14-322

**Published:** 2013-11-14

**Authors:** Christy C Tomkins-Lane, Lynne MZ Lafave, Jill A Parnell, Ashok Krishnamurthy, Jocelyn Rempel, Luciana G Macedo, Stephanie Moriartey, Kent J Stuber, Philip M Wilson, Richard Hu, Yvette M Andreas

**Affiliations:** 1 Department of Physical Education and Recreation Studies, Mount Royal University, 4825 Mount Royal Gate SW, Calgary, AB T3E 6K6, Canada; 2 Department of Nursing, Mount Royal University, 4825 Mount Royal Gate SW, Calgary, AB T3E 6K6, Canada; 3 Department of Physical Therapy, University of Alberta, 2-50 Corbett Hall, Edmonton, AB T6G 2G4, Canada; 4 Southport Atrium - Cubicle #1510, Alberta Health Services, 10301 Southport Lane SW, Calgary, AB T2W 1S7, Canada; 5 Canadian Memorial Chiropractic College, 19-8 Weston Drive SW, Calgary, AB T3H 5P2, Canada; 6 Department of Kinesiology, Brock University, WC25, 500 Glenridge Ave, St. Catharines, ON L2S 3A1, Canada; 7 Department of Surgery, University of Calgary, Room 0492, McCaig Tower, Foothills Medical Centre, 3134 Hospital Drive NW, Calgary T2N 5A1, Canada; 8 Office of Research Services, Mount Royal University, Mount Royal University, 4825 Mount Royal Gate SW, Calgary, AB T3E 6K6, Canada

**Keywords:** Lumbar spinal stenosis, Physical activity, Nutrition, Pedometer, Obesity, Exercise, Inactivity, Treatment

## Abstract

**Background:**

Because of symptoms, people with lumbar spinal stenosis (LSS) are often inactive, and this sedentary behaviour implies risk for diseases including obesity. Research has identified body mass index as the most powerful predictor of function in LSS. This suggests that function may be improved by targeting weight as a modifiable factor. An e-health lifestyle intervention was developed aimed at reducing fat mass and increasing physical activity in people with LSS. The main components of this intervention include pedometer-based physical activity promotion and nutrition education.

**Methods/Design:**

The Spinal Stenosis Pedometer and Nutrition Lifestyle Intervention (SSPANLI) was developed and piloted with 10 individuals. The protocol for a randomized controlled trail comparing the SSPANLI intervention to usual non-surgical care follows. One hundred six (106) overweight or obese individuals with LSS will be recruited. Baseline and follow-up testing includes dual energy x-ray absorptiometry, blood draw, 3-day food record, 7-day accelerometry, questionnaire, maximal oxygen consumption, neurological exam, balance testing and a Self-Paced Walking Test. Intervention: During Week 1, the intervention group will receive a pedometer, and a personalized consultation with both a Dietitian and an exercise specialist. For 12 weeks participants will log on to the e-health website to access personal step goals, walking maps, nutrition videos, and motivational quotes. Participants will also have access to in-person Coffee Talk meetings every 3 weeks, and meet with the Dietitian and exercise specialist at week 6. The control group will proceed with usual care for the 12-week period. Follow-up testing will occur at Weeks 13 and 24.

**Discussion:**

This lifestyle intervention has the potential to provide a unique, non-surgical management option for people with LSS. Through decreased fat mass and increased function, we may reduce risk for obesity, chronic diseases of inactivity, and pain. The use of e-health interventions provides an opportunity for patients to become more involved in managing their own health. Behaviour changes including increased physical activity, and improved dietary habits promote overall health and quality of life, and may decrease future health care needs in this population.

**Trial registration:**

Clinicaltrials.gov, NCT01902979

## Background

Lumbar spinal stenosis (LSS) is a degenerative condition which typically affects adults in their sixth and seventh decades of life [1]. The estimated prevalence of symptomatic LSS ranges between 8.4% [2] and 9.3% in the general population, [3] and is on the rise worldwide [4]. There are an estimated 1.2 million people in the US with symptomatic LSS at any given point in time [2].

LSS is characterized anatomically by a narrow spinal canal and/or narrow nerve root foramina, resulting from degenerative changes in the spine. The most specific symptom of LSS is neurogenic claudication, which includes pain, numbness and weakness in the low back, buttocks and legs brought on by standing and exacerbated by walking [5]. Because of these symptoms, people with LSS avoid walking and exhibit sedentary behaviour [6]. Therefore, not only is LSS painful and debilitating, it also has a considerable impact on risk for chronic diseases of inactivity.

Sedentary behaviour has important implications for overall health in the LSS population [7,8]. Physical inactivity and accompanying weight gain increase the risk of many chronic diseases, notably obesity, metabolic syndrome, coronary heart disease, type 2 diabetes, and certain cancers [9-22]. A growing body of literature also supports a link between obesity-related systemic inflammation and musculoskeletal pain [15-19,21,22]. In particular, relationships have been established between low back pain and both fat mass, [23] and body mass index (BMI) [24-30]. Specific to physical activity, a recent study identified BMI as the strongest predictor of day to day function in people with LSS [31]. BMI was a stronger predictor of function than both disease severity and pain. This suggests that by targeting weight as a modifiable factor in people with lumbar spinal stenosis, we may also impact function. It is possible that by decreasing fat mass and increasing physical activity, we can reduce systemic inflammation and decrease the risk of obesity-related diseases, including pain, in people with LSS.

Despite the potential implications of weight gain and inactivity in LSS, to our knowledge there has been no research to date examining non-surgical options for weight management in overweight and obese individuals with this condition. The present study aims to address this gap by evaluating a newly developed lifestyle intervention aimed at promoting weight loss in LSS through behaviour change techniques, pedometer-based physical activity promotion, and nutrition education. In keeping with recent trends in medicine, this program was developed using an e-health (online) platform. The use of e-health interventions provides an opportunity for patients to play an active part in management of their own health, in co-operation with healthcare providers. This intervention has the potential to provide a unique, non-surgical management option for people with LSS, decreasing the risk for obesity, chronic diseases of inactivity, and musculoskeletal pain through decreased fat mass and increased physical activity.

### Objective

The objective of this study is to evaluate an e-health lifestyle intervention aimed at increasing physical activity, decreasing fat mass, and increasing quality of life in overweight and obese people with LSS. The main components of this intervention consist of pedometer-based physical activity promotion and nutrition education.

## Methods/Design

The project consists of three phases. Details regarding Phases 1 (program development) and 2 (intervention pilot) have been submitted in a separate publication and will not be presented here.

### Phase 1, development (Complete)

Develop the online (e-health) intervention tool used to deliver weekly personalized pedometer step goals and tips, as well as nutrition education sessions.

### Phase 2, intervention pilot (Complete)

Over the past 2 years, the Spinal Stenosis Pedometer and Nutrition Lifestyle Intervention (SSPANLI) was developed and piloted with 10 overweight and obese individuals with LSS. This pilot data was used to evaluate the efficacy, content and feasibility of the intervention. Based on feedback obtained during post-pilot interviews, the intervention was shown to be feasible and attractive to participants. Statistically significant improvements were observed for fat mass (dual x-ray absorptiometry: DXA), trunk fat mass (DXA), physical function (accelerometry), symptom severity (Swiss Symptom Severity Scale), caloric intake (DA plus 10.0), and mental health (SF-36). Publication of the pilot data is forthcoming.

### Phase 3

The final phase in this project, described here, is a fully powered randomized controlled trial comparing the Spinal Stenosis Pedometer and Nutrition Lifestyle Intervention (SSPANLI) to usual care for lumbar spinal stenosis. Recruitment for the trial began in August 2013. Ethics approval for the trial was obtained through the Conjoint Health Research Ethics Board of the University of Calgary.

### Study design and overview

**Figure 1:**
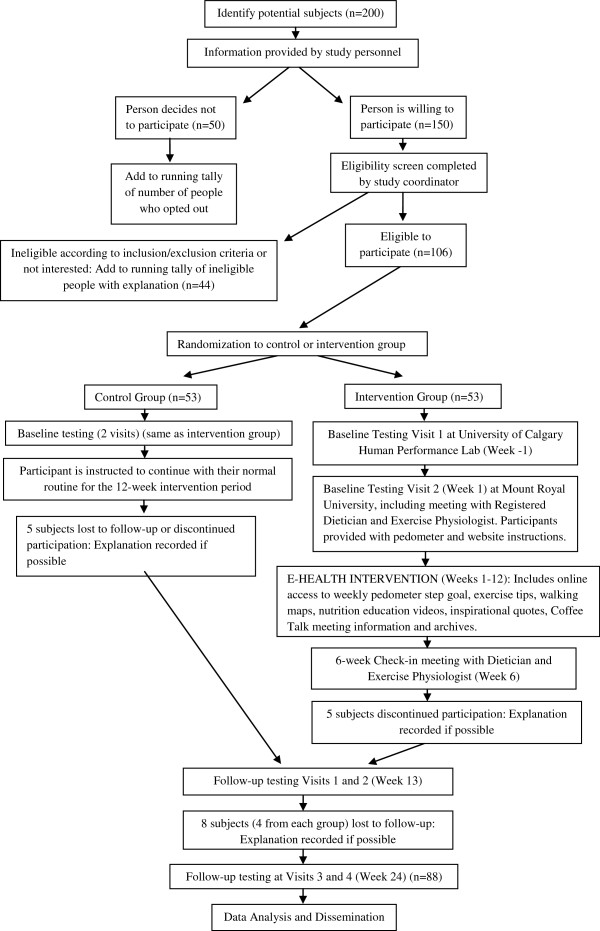
Study flowchart.

The Spinal Stenosis Pedometer and Nutrition Lifestyle Intervention (SSPANLI) is a single-blind RCT comparing the SSPANLI intervention to usual non-surgical care for LSS. This project will evaluate the new e-health lifestyle intervention aimed at decreasing fat mass, increasing physical activity and improving quality of life in older adults with LSS who are overweight or obese. The SSPANLI protocol includes individualized pedometer-based activity recommendations and online nutrition education tools, as well as two personalized consultations with a Registered Dietitian and exercise specialist. Figure 1 presents a flowchart for the study.

#### Inclusion and exclusion criteria

All participants will be at least 45 years of age and will have received a diagnosis of LSS by a spine surgeon who has both examined the patient and reviewed imaging results (magnetic resonance imaging or computed tomography). All participants will be required to have a BMI of 25 kg/m^2^ or greater (overweight) and to have maintained a stable bodyweight for the previous 3 months. Exclusion criteria include diagnosed eating disorders, pregnancy, weight over 350 lbs, or any co-morbid conditions that would limit walking significantly or make participation in a walking program medically inadvisable, including severe arthritis, neuropathy or other neuromuscular disease, angina, cardiovascular disease, pulmonary disease, stroke or other neurological disorder, or peripheral vascular disease. Participants currently participating in a diet or lifestyle intervention for weight loss, who are on medications or herbal preparations known to influence bodyweight (including, but not limited to antidepressants and orlistate), or who have had changes in their prescription of lipid-lowering or diabetes medications over the past 3 months will be excluded.

#### Recruitment

All participants will be recruited through the University of Calgary Spine Program and Caleo Health. Spine patients are referred to Caleo Health Centre for a complete spine assessment to determine the most appropriate treatment (referral to a surgeon, physical medicine, physiotherapy, combination therapy etc.). It is through this triage process that the research nurse will be able to identify potential participants and contact them for possible participation in the trial. Spine surgeons from the University of Calgary Spine Program will also be made aware of this protocol, so that that their patient populations can be reviewed for potential inclusion. In the triage process, a review of the medical history including height and weight will be obtained (used to determine BMI). This information will be documented on both a triage form as well as documentation provided to the referring doctor. This information will be reviewed by the research nurse (who currently reviews all triage forms). Potential patients will either be contacted by phone or seen the day of the clinic visit to discuss the study. A screening form will be used to identify potential participants based on inclusion/exclusion criteria.

### Blinding and randomization

After completing the screening process participants will be randomly assigned to either the intervention group (n = 53) or the control group (n = 53). A computer-generated randomization sequence produced using R statistical analysis program will be used. Participants will be allocated to the group by the Study Coordinator, and will be aware of the allocated group. Those assigned to the control group will be offered the opportunity to try the intervention once the study is complete. All study investigators including the statistician, and research staff collecting data will be blinded to group allocation (single blind design). Participants will be asked not to comment to assessors regarding allocation.

### Testing schedule

Following enrollment, patients will begin the study protocol, which involves 7 testing appointments over a 6 month period. These appointments include Baseline Visit 1 (week -1), Baseline Visit 2 (week 1), 6-week check-in (week 6), Follow-up Visits 1 and 2 (week 13) and Follow-up Visits 3 and 4 (week 24). The intervention begins at Baseline Visit 2 and runs for 12 weeks (weeks 1–12). Enrollment will be continuous throughout the study. Once patients are enrolled they will begin assessments at the next available scheduled date for Baseline Visit 1. Six-week check-in sessions will be arranged individually for each participant. The primary outcomes at each time point will be fat mass (%), steps/day, and quality of life (SF-36).

### Baseline testing and intervention

Baseline testing will take place over two visits. During the second baseline visit, components of the intervention will be introduced.

### Baseline visit 1

During Baseline Visit 1, anthropometric measurements of height, weight and waist circumference will be taken. To assess body composition participants will undergo dual energy x-ray absorptiometry (DXA) testing. The DXA scan uses a low emission x-ray in conjunction with Hologic software to provide measurements for lean mass, fat mass and bone mineral density (Hologic QDR 4500, Hologic, Inc., Bedford, MA).

#### Blood collection

All blood draws will be performed after an 8 hour overnight fast by a trained nurse or phlebotomist. 2 ml of blood will be collected with sodium fluoride for glucose analysis. A further 3 ml of blood will be collected in serum tubes for adipokine analysis (adiponectin, c-reactive protein (CRP), leptin, IL-1β, IL-6, IL-8, TNF-α, MCP-1 and insulin). A final 4 ml will be collected for serum lipid analysis. Samples will be centrifuged and stored at -80°C until analysis.

#### Plasma glucose

Blood glucose concentrations will be measured in triplicate using a glucose trinder assay kit (Stanbio Laboratory, Boerne, TX). Serum Adipokines: Adiponectin will be measured in duplicate using an ELISA kit from Millipore (Millipore, Billeerica, MA). For leptin, IL-1β, IL-6, IL-8, TNF-α, MCP-1 and insulin duplicates samples of 25 μl of serum will be analyzed using a Milliplex MAP Human Serum Adipokine Panel B kit (Millipore, Billerica, MA). CRP will be measured by Calgary Laboratory Services (Calgary, AB). Serum lipids: 1.5 ml of serum will be sent to Calgary Laboratory Services for quantification (total cholesterol, HDL cholesterol, LD cholesterol and triglycerides will be calculated).

#### 7-day activity monitoring

Participants will be provided with an activity monitor (Actigraph GT3X, Actigraph LLC, Pensacola, FL.) and an activity log. Participants will be instructed to wear the monitor during the waking hours, except when in contact with water. Participants will also be instructed how to complete a daily log to accompany the activity monitor. Participants will be asked to wear the monitor for the 7 days following Baseline Visit 1, to establish baseline activity level (steps per day). The activity monitor can provide an objective record of frequency, intensity, and duration of physical activity with minimal burden on participants. The Actigraph is widely accepted in the field as a valid and reliable means of assessing ambulatory physical activity [32-36].

#### 3-day food records

Food and beverage intake will be assessed using 3-day food records. Participants will be trained on how to complete a 3-day food record by a Registered Dietitian on their first visit. Participants will then be asked to estimate portion sizes and record all food and beverages consumed for 2 weekdays and 1 weekend day. The food record will be completed in the week following Baseline Visit 1, to establish baseline dietary intake. Dietary intakes will be analyzed with Diet Analysis Plus 10.0 software (Thomson Wadsworth, Toronto, ON).

Participants will be provided with a self-addressed, stamped envelope to return the activity monitor, activity log, and 3-day food record to Mount Royal University.

### Baseline visit 2

Baseline testing during Baseline Visit 2 will include questionnaire completion, a Self-Paced Walking Test, balance testing, a neurological examination, and peak VO_2_ exercise test on a cycle ergometer. Intervention components of Baseline Visit 2 will include a one hour personalized consultation session with a Registered Dietitian, and a one hour personalized physical activity counseling session with an Exercise Physiologist (including instructions regarding how to use the pedometer). Participants will also be guided through the process of initial login and navigation of the e-health intervention website, as well as provided with instructions regarding how to fill out the daily personal log book.

#### Baseline testing details

##### Questionnaire

The questionnaire will be used to collect information regarding age, gender and history of back and leg pain, as well as history of medications, alcohol use, tobacco use and current work status. The standardized instruments to be used can be found in Table 1. The questionnaire will be completed online through the intervention website.

**Table 1:** Standardized questionnaires

**Questionnaire**	**Description**
Physical Function Scale of Swiss Spinal Stenosis Questionnaire (PF Scale) [1]	The Physical Function Scale was designed to assess walking capacity in people with LSS. The score is calculated as the un-weighted mean of the five items in the scale. The resulting possible scores of 1–4 represent a range from mild to severe limitation in physical function/walking.
Symptom Severity Scale of the Swiss Spinal Stenosis Questionnaire (SS Scale) [1]	The Symptom Severity Scale of the SSSQ was designed to examine severity of symptoms related to LSS. It is scored as the un-weighted mean of the seven items in the scale, with scores from one to seven representing a range from mild to very severe pain.
Oswestry Disability Index (ODI) [37]	The ODI is a nine-item questionnaire, which assesses degree of back pain–related disability. Severity of pain and disability in activities, such as walking, sitting, standing, and personal care, are rated on Likert scales of five or six points. The ODI was calculated as a percentage of the total possible score of 53, with a greater score representing greater back pain–related disability.
Short-Form 36 (SF-36) [38]	Overall health and disability will be assessed using the 36-Item Short Form Health Survey. Using the 36 items, the following scales are calculated: General Health, Physical Functioning, Social Functioning, Role Limitation—Physical, Role Limitation—Emotional, Mental Health, Vitality, and Bodily Pain. Each scale score is calculated independently using algorithms defined by the developers.
Centres for Disease Control Depression Scale (CES-D) [39]	The 20 item Centres for Epidemiologic Studies Depression Scale was designed to assess depression. It is scored by totaling all item scores, with a higher total indicating greater depression. Scores range from 0 to 60, with higher scores indicating more symptoms of depression. CESD scores of 16 to 26 are considered indicative of mild depression and scores of 27 or more indicative of major depression.
Regulation for Eating Behaviors Scale (REBS) [40]	To assess healthy eating motives, participants will complete the 24-item Regulation of Eating Behaviour Scale. Six subscales comprised of 4 items/subscale formulate the REBS including (a) Amotivation, (b) External Regulation, (c) Introjected Regulation, (d) Identified Regulation, (e) Integrated Regulation, and (f) Intrinsic regulation. Participants respond to each REBS item on a 7-point Likert scale anchored at the extremes by (1) 'Does not correspond at all’ and (7) 'Corresponds exactly’.
Behavioral Regulation in Exercise Questionnaire 2-R (BREQ-2R) [41,42]	•To assess exercise motive related to exercise, participants will complete the 19 item Behavioral Regulation in Exercise Questioniare-2 plus four items assessing Integrated Regulation. The BREQ-2 assesses the following constructs: (a) Amotivation, (b) External Regulation, (c) Introjected Regulation, (d) Identified Regulation, and (e) Intrinsic Regulation. Reponses to the BREQ-2 and Integrated Regulation items were made using a 5-point Likert scale anchored at the extremes by 0 ('Not true for me’) and 4 ('Very true for me’).
Pain Catastrophizing Questionnaire [43]	The PCS was designed to assess pain catastrophizing. The PCS is a 13-item instrument that asks participants to reflect on past painful experiences, and to indicate the degree to which they experience each of 13 thoughts or feelings when experiencing pain, on 5-point scales with the end points (0) not at all and (4) all the time. The PCS total score is computed by summing responses to all 13 items. PCS total scores range from 0 – 52. A score of 30 or above represents clinically relevant catastrophizing.
Tampa Scale for Kinesiophobia (TSK) [44,45]	The TSK was designed to assess fear of movement (kinesiophobia). The TSK uses a 4-point Likert scale, with scoring options ranging from 1 = 'strongly disagree’ to 4 = 'strongly agree’. A total score is calculated after inversion of the individual scores of items 4, 8, 12 and 16. The total score ranges between 17 and 68. A high value on the TSK indicates a high degree of kinesiophobia. A score of 37 differentiates between high and low scores.

##### Self-paced walking test

All participants will undergo a standardized Self-Paced Walking Test [46]. This test has been shown to be a valid criterion measure of walking capacity in people with LSS. Participants are instructed to walk continuously at their own pace until they feel that they have to stop due to symptoms of LSS (or other reasons) or until a time limit of 30 minutes has been reached. Participants are asked to indicate when they first experience a change in symptoms, as well as to indicate the nature of the symptoms (type and location). Test termination is defined as a complete stop of 3 seconds or more. The following information is collected: total distance and time walked, time/distance to onset of symptoms, the nature and location of symptoms (pain, numbness/tingling, weakness or fatigue), average walking speed, and the reason for test termination should they not walk for the full 30 minutes (symptoms of LSS, fatigue, shortness of breath, dizziness, pain or discomfort due to co-morbidities). Before and immediately after the walking test, participants will be asked to rate their current pain on 100 mm Visual Analog Pain Scales for back, right leg and left leg, with anchors of 0 (no pain) and 10 (worst possible pain). VAS scales have been used often in research with spinal stenosis patients and found to be psychometrically sound [47,48]. Participants will also be asked to indicate on a body diagram the location and nature of their symptoms.

##### Balance testing

Balance will be assessed using a HUR-3 Balance platform. Each participant will be assessed for 20 seconds on each of the following stances (all with eyes open and a firm surface): double leg stance, tandem stance with right leg forward, tandem stance with left leg forward, single leg right, single leg left. For each test, participants will be asked to place their hands on their hips and face forward. Balance test results are stored using the HUR-3 software.

##### Neurological impairment score [49]

Both lower limbs are tested and five parameters are assessed. Points are added up to a maximum total score of 33 (with a higher score indicating less neurological impairment). 1. Tendon (myotatic) reflexes in lower limbs, that is, patellar reflex and Achilles tendon reflex. For each reflex evoked (using facilitating manoeuvres), the person examined is given 1 point, potential score 0–4 points. 2. Tactile sensation in lower limbs using a cotton swab. If there is a dysfunction of tactile sensation in both lower limbs, the person examined receives 0 points. If there is a dysfunction of tactile sensation in only one lower limb then the score is 2 points. If tactile sensation is bilaterally normal, the score is 4 points. 3. Vibration sensation in the lower limbs will be assessed using a 128-Hz graduated tuning fork for optimum accuracy. The vibration threshold is recorded on a scale from 0 to 8, with the maximum expressed as 8/8 and minimum 0/8. Vibration sensation will be recorded at the external ankle. 4. Presence of paresis of lower limbs will be assessed by asking the participant to stand on tiptoe, stand on the heels, and squat. Each exercise successfully performed scored 1.5 points (giving a total range of 0–9 points). Squatting is considered as the ability to stand from a full squatting position. Whenever the results of these tests are in doubt (e.g., where the influence of pain or joint arthrosis appeared possible), the presence of paresis will be verified by isometric muscle testing. 5. Evaluation of ability to walk and run. If the participant is able to run at least 10 m, 12 points will be awarded. If the person is able to walk this distance without support he/she receives 9 points. Walking with one crutch will score 6 points, walking with the aid of two crutches will score 3 points. If the participant is unable to walk, the score will be 0.

##### VO_2peak_ testing protocol

The test administrator will review the participant’s medical history, physical findings and check for any absolute contraindications to exercise, or signs indicative of major cardio-vascular, pulmonary or metabolic disease. All tests will be conducted by a Certified Exercise Physiologist or Kinesiologist. Prior to exercise testing, height and weight will be measured, and 12-lead electrocardiogram (ECG) electrodes placed. The BORG scale for perceived exertion (RPE) will be explained to the participant [50]. Resting heart rate, blood pressure and ECG readings will be obtained over 4 minutes prior to testing [51-54]. The participant will be set up at the appropriate seat height on an electrically braked cycle ergometer. A face mask will be connected to a metabolic cart for breath by breath gas analysis. The ECG will remain connected to a three channel recorder for ECG, time and ventilation recordings [55]. The following variables will be recorded in the last 15 seconds of each minute during 4 minutes of unloaded cycling, before and after the test, and during the last 15 seconds of each stage in the incremental protocol to follow: heart rate, ventilation, volume of oxygen (VO_2_), volume of carbon dioxide, and respiratory exchange ratio (RER) [51,56]. Blood pressure will be measured before and after the test, as well as in the last 15 seconds of each third minute [54]. RPE will be measured in the last 15 s of each third minute. Throughout the test the ECG will be monitored for abnormal changes.

The individualized incremental protocol to be used is taken from Wasserman et al. [56] and has been found to produce accurate estimates of VO_2 peak_[56]. For each participant, VO_2_ unloaded is calculated from the participant’s weight, and VO_2peak_ is estimated from the participant’s age and height [56]. The work rate increment necessary to reach the estimated VO_2peak_ in 10 minutes is calculated. This time limit is chosen because it has been shown that incremental tests of 6–12 minutes provide the highest VO_2peak_ for normal participants [56].

1. VO_2_ unloaded in ml/min = 150 + (6 × weight in kg)

2. VO_2_ peak in ml/min = (height in cm ‒ age in years) × 20 for sedentary men and × 14 sendentary women

3. Work rate increment per minute in watts = (VO_2_ peak estimate ‒ VO_2_ unloaded estimate)/100

After 4 minutes of unloaded cycling, the calculated work load is applied increased by that amount every minute until a symptom limited test endpoint is reached. Participants will be encouraged to continue until they feel that for whatever reason, they are unable to. Once the test is terminated, the participant will continue unloaded cycling for 4 minutes. The participant will be asked to indicate what symptoms caused he/she to stop the test [56]. All variables will continue to be measured as described during this time. The ECG, blood pressure and RPE will be monitored every minute until values return to baseline [52].

VO_2peak_ will be determined as the highest value for VO_2_ averaged over a 30 second period [54], and will be confirmed by any of the following criteria [52]: Failure of heart rate to increase with increasing load, or achievement of 85% age predicted max heart rate; a plateau in oxygen uptake with increasing work-load; RER of >1.15; RPE > 17 on the BORG scale.

#### Intervention component details

Components of the intervention can be found in Table 2.

**Table 2:** Intervention components

**Intervention component**	**Purpose**
Registered Dietitian Consultations	Provides personalized nutrition education and behaviour modification strategies related to dietary intake
Exercise Physiologist Consultations	Provides physical activity education and behaviour modification strategies related to physical activity
Personalized step goal and physical activity tips	Provides motivation to increase physical activity
Daily wear of pedometer	Provides opportunity for self-management and bio-feedback
Nutrition education videos	Provides nutrition education for self-management
Log book	Provides opportunity for self-management
Coffee Talk	Provides social context and peer support

#### Nutrition counseling and education component

During Baseline Visit 2, each participant will meet with a Registered Dietitian for an initial nutrition counseling session of approximately 1 hour. This session will involve a review of the baseline 3 day food record, suggestions targeting reduced energy intake, and counseling on behaviour change strategies including goal setting, self-monitoring, stimulus control, and social support [57]. Each participant will be provided with methods for 454 a daily caloric reduction of 500–750 kcal. Each week, participants will log on to the intervention website to access new nutrition education sessions directed at supporting improved nutrition behaviors, weight loss, and behaviour change.

#### Physical activity counseling session and promotion component

During Baseline Visit 2, participants will meet with an exercise specialist to discuss goals, barriers to physical activity, personal strategies for increasing activity, and walkability of the participant’s neighbourhood. They will discuss the results of their VO_2_ test in relation to goal setting. Each participant will then receive a pedometer and a personalized daily step goal for Week 1. Participants will wear the pedometer daily, during the waking hours. Each subsequent week, participants will log on to the intervention website to receive a new individualized daily step goal. Participants will be instructed to attempt to meet the step goal in whatever way they feel capable, and will be provided with new 'tips’ each week. This method is thought to be well suited to LSS patients, as opposed to traditional time or distance goals for walking, given that participants can accumulate steps in small bouts throughout the day and within their homes. The goal will be to increase steps per day by a total of 20% by the end of the 12 week intervention period. The goal will be calculated as an increase of 1.3% above baseline for weeks 1–6 and 2% for weeks 7–12.

#### Intervention website training

During Baseline Visit 2, each participant will be guided through the login process for the intervention website, as well as instructed regarding how to navigate the website. During the initial registration, participants will enter their baseline daily step count, as determined from the 7-day activity monitoring. This value will be used to calculate the personalized weekly step goals.

#### Log book training

During Baseline Visit 2, each participant will be instructed regarding how to fill out the daily log book. Over the 12 week intervention period participants will record the following daily: food and water intake, physical activity: daily steps as well as duration (minutes) and intensity (mild, moderate or severe) of any structured activity, sleep (hours), mood, and medications.

#### 12- Week Intervention (at participants’ home)

Starting the day of Baseline Visit 2, each week for 12 weeks participants will log on to the e-health intervention website to access weekly inspirational quotes, their personal weekly physical activity goal (steps) and tips regarding how to achieve this goal. Each week they will also access a nutrition education video. The nutrition video topics are as follows: Weight loss – secrets to success; Meal timing and tracking; The art of snacking; Environment for success; Food diaries; Feeling full with fibre and hydration; Be a fat and calorie detective; Saboteurs to weight loss parts 1 and 2; Energy density; Mindful eating; and The 10 habits of successful weight loss maintainers. Every three weeks there will be an optional 'Coffee Talk’ meeting. These meetings will be casual gatherings meant to provide social context and support to participants. A member of the study staff will be present at each Coffee Talk to discuss progress and ideas. These meetings will be announced through the intervention website. At any time, participants will be able to access content from previous weeks via the archives.

#### 6-Week check-in session

The 6-week Check-in session will be at mid-intervention. Participants will meet with the Registered Dietitian and Exercise Physiologist for a second time to discuss individual progress, barriers to success and suggested modifications.

#### Follow-up

There will be two follow-up testing time points. The first will be directly following the intervention at 13 weeks and the second at 24-weeks. Protocols for follow-up testing will be identical to baseline testing with two visits at each time point. Each follow-up will include a visit to the University of Calgary Human Performance Lab as well as a visit to Mount Royal University Exercise Sciences Lab. At the Human Performance Lab participants will have DXA testing, anthropometric measurements, a blood draw, and instructions regarding how to complete the final 7-day activity monitoring and 3 day food record. At the Exercise Sciences Lab, participants will complete the follow-up questionnaire, Self-Paced Walking Test, balance testing, neurological impairment score and VO_2_ peak testing. The questionnaire will be identical to the baseline questionnaire, with the addition of the change questions. The change questions are on 7-point likert scales and assess perceived change in activity level, mood, overall health, calorie intake, vegetable intake and fibre intake.

All participants will be invited back one month after each follow-up testing session to review their results with study personnel. Those assigned to the control group will be offered the opportunity to try the intervention once the study is complete.

### Magnetic resonance imaging

Following the 12-week intervention period for each participant, we will access the clinical magnetic resonance imaging (MRI) report. The variables to be extracted from the MRIs include dural cross sectional area at each lumbar level, as well as cross-sectional area of the paraspinal muscles (psoas and multifidus).

### Sample size estimate

Pilot study data was used to power the planned RCT. The pilot study provided an estimate of the variability in the number of steps taken for 10 participants with LSS. We used a crude estimate of the sample standard deviation for steps in place of the unknown population standard deviation. Our main study outcome is number of steps at the end of the 12-week intervention. For the pilot study, an average increase of 375 steps was encouraged during each six-week period. At the end of the study, an average increase of 750 steps was anticipated. This was used to judge whether the intervention was effective. With α = 0.05 and a mean difference of 750 steps at the end of study, the standard deviation of the difference was calculated as follows:

$$\mathrm{Std}.\mathrm{Dev}\;\mathrm{of}\;\mathrm{diff}=\tilde{\sigma }=\frac{\left(L-S\right)}{4}=\frac{\left(5000-0\right)}{4}=1250$$

L equals largest and S equals smallest anticipated difference in the number of steps from baseline to follow-up period.

The formula for the sample size required, per group, (with α = 0.05, the probability of type I error, 80% power and an effect size of 0.6) to compare two population means, *μ*_1_ and *μ*_2_ with common variance, *σ*^2^, is

$$n\;=\;\frac{2{\left(Z_{1-\alpha /2}+Z_{1-\beta }\right)}^{2}}{{\left(\frac{\mu_{1}-\mu_{2}}{\sigma }\right)}^{2}}=\;\frac{2{\left(1.96+0.84\right)}^{2}}{{\left(\frac{750}{1250}\right)}^{2}}=43.60\approx 44$$

We concluded that a sample size of 44 people per group would be needed to detect a between-group difference of number of steps taken from baseline to week twelve. Based on the dropout rate for the pilot, we anticipate a potential dropout rate of 20% for the RCT. We will therefore aim to recruit 106 participants (53 per group). This number has been deemed feasible based on the volume of lumbar spinal stenosis patients seen through the University of Calgary Spine Program (approximately 500 per year).

### Statistical analysis

Using the raw quantitative data from the participants of both groups we will present descriptive statistics summarizing the mean and standard deviation with respect to all the primary study variables. Age and sex distributions of the participants will be tabulated. Our goal is to establish reliable point estimates of the study variables and to characterize the variability within the two groups.

The independent variable will be the intervention, while the dependent variables will include steps/day, fat mass (%) and health related quality of life (SF-36 overall score). Linear mixed-effects models will be used to determine whether the changes for each time period (baseline to 13 weeks, baseline to 24 weeks) were significantly different between intervention and control groups. Repeated measures analysis of variance (RANOVA) will be used to examine within group changes from baseline to 13 weeks and from baseline to 24 weeks.

We will also examine changes in the following variables: Biochemical markers of obesity and associated co-morbidities including Plasma glucose, Serum adipokines (c-reactive protein (CRP), leptin, IL-1β, IL-6, IL-8, TNF-α, MCP-1 and insulin), and Serum lipids (total cholesterol, LDL cholesterol, HDL cholesterol and triglycerides); Physical function (Physical Function Scale of Swiss Spinal Stenosis Questionnaire); Walking capacity (Self-Paced Walking Test distance); Balance (mean deviation from centre of pressure from balance platform); Neurological impairment (Neurological Impairment Score); Pain (Symptom Severity Scale and visual analog scales); Back pain related disability (Oswestry Disability Index); Fear of movement (Tampa Scale for Kinesiophobia); Depression (CES-D total score); kilocalorie intake (kcal/day); Fat intake (grams/day); Vegetable and fruit intake (Canada Food Guide servings/day); Fibre intake (grams/day); and Change questions for activity, mood, overall health, calories, vegetable intake, and fibre intake. Clinical MRIs we will be used to assess whether imaging findings are predictive of change in the primary outcome variables following the intervention.

When an inferential test is performed, the level of significance, α will be set at 0.05, and results will be presented with p-values. In all tests, p-values less than 5% (< 0.05) will be considered statistically significant. All quantitative analyses and hypothesis will be performed using Minitab software, version 16 ^5^ and R programming language, version 2.15.1. Specifically, we will use the *gee* package, *pwr* package and *samplesize* package downloaded from the Comprehensive R Archive Network (CRAN) repository for our power and sample size calculations.

## Discussion

The estimated cost of LSS in the US exceeds 2 billion dollars annually, with an estimated 2-year cost of $10,486 per person for non-surgical care, and $88,000 per surgery [58,59]. There is therefore a need to develop novel and effective treatments for individuals with this condition [60,61]. Owing to age and symptom-related mobility limitations, people with LSS are at risk for sedentary behavior and obesity. Yet, there has been no research to date examining options for weight management in individuals with LSS. It is proposed that weight loss could be accomplished in this population using a recently developed patient-centered e-health lifestyle modification approach of physical activity promotion and nutrition education (SSPANLI).

The use of e-health interventions provides an opportunity for patients to become more involved in managing their own health and wellness. Lifestyle behaviour changes including increased physical activity, and improved dietary habits promote overall health and may decrease future health care needs. Ultimately, improved overall health will lead to increased functional autonomy and quality of life. In LSS specifically, weight loss and increased physical activity can minimize the need for expensive interventions (e.g. injection and surgery), improve mobility, and reduce the risk of avoidable health deterioration. It is possible that by increasing physical activity and decreasing fat mass, we can modify the risk for diseases of inactivity, including systemic inflammation, metabolic syndrome, type 2 diabetes, cardiovascular disease. Through reduction of fat mass we may also reduce the risk for musculoskeletal pain expression and chronicity. Further, this intervention will support individuals who require surgery in attaining their recommended pre-surgical weight, as well as reduce the risk of complications, and revision surgeries. This study is significant as it represents the first time an online platform would be used to deliver a lifestyle intervention aimed at managing LSS and risk for diseases of inactivity concurrently. Because the results of the SSPANLI pilot suggest that this intervention is feasible, attractive to individuals with LSS, and effective in a small non-randomized sample, SSPANLI has the potential to offer an effective, inexpensive, and non-invasive treatment option for the millions of North Americans living with LSS today [3].

## Abbreviations

LSS: Lumbar spinal stenosis; BMI: Body mass index; DXA: Dual x-ray absorptiometry; SSPANLI: Spinal stenosis pedometer and nutrition lifestyle intervention; SF-36: Short Form 36 Questionnaire; ECG: Electrocardiogram; RPE: Rating of perceived exertion; VO2: Volume of oxygen; MRI: Magnetic resonance imaging; CRP: C-Reactive protein.

## Competing Interests

The authors declare that they have no competing interests.

## Authors’ contributions

CTL conceived and designed the study, acquired, analyzed an interpreted pilot data, and drafted all versions of the manuscript. LL, JP, JR, SM, KS and RH contributed to design of the study, acquisition and interpretation of pilot data, and critical revision the manuscript. LM and AK were responsible for statistical analysis of pilot data and preparation of statistical analysis plans for the protocol. All authors read and approved the final manuscript.

## Pre-publication history

The pre-publication history for this paper can be accessed here:

http://www.biomedcentral.com/1471-2474/14/322/prepub
